# Aboriginal and non-Aboriginal children in Western Australia carry different serotypes of pneumococci with different antimicrobial susceptibility profiles

**DOI:** 10.1186/s41479-016-0015-9

**Published:** 2016-09-05

**Authors:** Eileen M. Dunne, Kylie Carville, Thomas V. Riley, Jacinta Bowman, Amanda J. Leach, Allan W. Cripps, Denise Murphy, Peter Jacoby, Deborah Lehmann, the Kalgoorlie Otitis Media Research Project Team

**Affiliations:** 1 Pneumococcal Research, Murdoch Childrens Research Institute, Parkville, VIC Australia; 2 Victorian Infectious Disease Reference Laboratory at the Peter Doherty Institute for Infection and Immunity, Melbourne, VIC Australia; 3 Microbiology & Immunology, School of Pathology & Laboratory Medicine, The University of Western Australia, Crawley, WA Australia; 4 Department of Microbiology, PathWest Laboratory Medicine, Queen Elizabeth II Medical Centre, Nedlands, WA Australia; 5 Child Health Division, Menzies School of Health Research, Darwin, NT Australia; 6 School of Medicine and Menzies Health Institute Queensland, Griffith University, Southport, QLD Australia; 7 Public Health Bacteriology Laboratory, Centre for Public Health Sciences, Coopers Plains, QLD Australia; 8 Telethon Kids Institute, The University of Western Australia, Subiaco, WA Australia

**Keywords:** *Streptococcus pneumoniae*, Carriage, Serotypes, Pneumococcal conjugate vaccine, Aboriginal

## Abstract

**Background:**

Carriage of *Streptococcus pneumoniae* is considered a precursor to pneumococcal diseases including pneumonia. As part of the Kalgoorlie Otitis Media Research Project, we characterised pneumococci isolated from the nasopharynx of Western Australian Aboriginal and non-Aboriginal children.

**Methods:**

Between 1999 and 2005, 100 Aboriginal and 180 non-Aboriginal children were followed from birth to two years, with nasopharyngeal aspirates collected at ages 1–3 and 6–8 weeks, then at 4, 6, 12, 18 and 24 months. Introduction of 7-valent pneumococcal conjugate vaccine (7vPCV) in 2001 enabled evaluation of its impact on carriage in study participants according to vaccines doses received. Pneumococcal serotyping was performed by Quellung and antimicrobial susceptibility by disk diffusion and Etest®. Molecular epidemiology of pneumococcal isolates was investigated by pulse-field gel electrophoresis and multilocus sequence typing.

**Results:**

Overall, the prevalence of 7vPCV serotypes was similar for Aboriginal and non-Aboriginal children (19 % vs. 16 %), but the prevalence of non-vaccine serotypes was higher in Aboriginal children (22 % vs. 7 %). A multi-resistant 6B clone (ST90) was found only in non-Aboriginal children. Aboriginal children who received three doses of 7vPCV had lower odds of carrying 7vPCV serotypes (odds ratio [OR] 0.19, 95 % CI 0.08–0.44) and higher odds of carrying non-vaccine serotypes (OR 2.37, 95 % CI 1.13–4.99) than unvaccinated Aboriginal children; this finding was not observed in non-Aboriginal children.

**Conclusions:**

This unique study shows important differences in pneumococcal serotypes, genotypes, and antimicrobial susceptibility between Aboriginal and non-Aboriginal children living in the same geographic area before widespread 7vPCV use, and highlights the need for ongoing post-vaccination surveillance in outback Australia.

**Electronic supplementary material:**

The online version of this article (doi:10.1186/s41479-016-0015-9) contains supplementary material, which is available to authorized users.

## Background

Pneumonia is a major cause of childhood morbidity and mortality worldwide and is estimated to be the second leading cause of death for children under the age of five, behind preterm birth complications [1]. Although the majority of disease burden occurs in Africa and south Asia, indigenous peoples living in high-income countries also have high rates of pneumonia [2, 3]. *Streptococcus pneumoniae* (the pneumococcus) is the predominant cause of community-acquired pneumonia [4, 5]. Pneumococci commonly reside in the nasopharynx of healthy young children [6, 7], and there are links between pneumococcal colonisation and invasive pneumococcal disease, pneumonia, and otitis media (OM) [8–10]. Studies in Australia have reported higher rates of pneumococcal carriage and disease in Aboriginal than in non-Aboriginal populations, and pneumococcal carriage occurs earlier in Aboriginal children [11–13]. However, there are no comparative data on pneumococcal serotypes and antimicrobial susceptibility in Aboriginal and non-Aboriginal children living in the same geographic region, either in Australia or elsewhere.

The pneumococcus is a highly diverse species, with variations in capsule structure giving rise to over 90 known serotypes. The use of pneumococcal conjugate vaccines (PCVs) in children can prevent invasive pneumococcal disease [14, 15] and OM [16, 17] caused by vaccine serotypes, as well as pneumonia [18, 19]. PCVs also reduce carriage of vaccine serotypes, but typically this reduction is associated with increased carriage of non-vaccine serotypes [6, 7, 20–22]. 7vPCV (which includes serotypes 4, 6B, 9V, 14, 18C, 19F and 23F) was included in the Australian immunisation schedule in 2001 for Aboriginal and high risk children, and in 2005 for all other children. This was replaced by PCV13 (7vPCV + serotypes 1, 3, 5, 6A, 7F, 19A) in 2011 [23].

As part of an investigation into causal pathways to OM, the Kalgoorlie Otitis Media Research Project examined bacterial carriage in young Aboriginal and non-Aboriginal children in the Kalgoorlie–Boulder area, Western Australia [11, 24]. Pneumococcal carriage rates were high in Aboriginal children: 18 % in the first month of life, and ranging between 51 and 67 % from age 3 months onwards. Equivalent figures for non-Aboriginal children were 3 % and 26–37 %, respectively [11]. The number of children in the household was positively associated with pneumococcal carriage for both Aboriginal and non-Aboriginal children, and day care attendance was a risk factor for carriage in non-Aboriginal children [25]. Here, our aim is to describe the serotypes, antimicrobial susceptibility, and molecular epidemiology of pneumococci isolated from the nasopharynx during the Kalgoorlie Otitis Media Research Project and the impact of 7vPCV on upper respiratory tract carriage among participants in this study. We hypothesise that there are differences in the serotypes and characteristics of pneumococci carried by Aboriginal and non-Aboriginal children.

## Methods

### Study design

The design of the Kalgoorlie Otitis Media Research Project has been described elsewhere [11, 24]. Briefly, between April 1999 and January 2003, 100 Aboriginal and 180 non-Aboriginal children residing in the Kalgoorlie–Boulder area were enrolled soon after birth. Written informed consent was provided by participants’ guardians. Nasopharyngeal aspirates (NPAs) were collected at ages 1–3 and 6–8 weeks, and at 4, 6, 12, 18 and 24 months as previously described [11]. NPAs were added to 1 ml skim milk-tryptone-glucose-glycerol broth and placed immediately at −20 ° C until transfer to −70 ° C within 72 hours. NPAs were collected, rather than nasopharyngeal swabs, to facilitate viral as well as bacterial detection, and have been shown to have similar pneumococcal isolation rates to nasopharyngeal swabs [26]. In July 2001, 7vPCV was introduced into the routine immunisation schedule for all Australian Aboriginal children to be given at ages 2, 4 and 6 months with a catch-up program for all Aboriginal children aged <2 years. A booster of 23-valent pneumococcal polysaccharide vaccine (23vPPV) was recommended for Aboriginal children at age 18 months. In the absence of a universal 7vPCV program for non-Aboriginal children during the study period, from October 2001 we also offered the same 3-dose schedule of 7vPCV to non-Aboriginal study participants. Vaccination characteristics of the study population are shown in Additional file 1: Table S1.

### Primary culture and serotyping of pneumococci

Selective media were used to isolate *S. pneumoniae* [11] and 4 colonies, preferably morphologically different colonies, were subcultured. Serotyping using the Quellung reaction was performed on all 4 subcultured colonies, with any identified serotypes reported once per sample and defined as ‘distinct pneumococcal isolates’.

### Antimicrobial susceptibility

Antimicrobial susceptibility testing was performed on one subcultured pneumococcal isolate from each sample, and also on any additional serotype or morphologically distinct isolate identified in the sample. We used disc diffusion [27] and the minimum inhibitory concentration (MIC) for penicillin- or ceftriaxone-resistant strains determined by Etest® (bioMerieux, USA) according to the recommended breakpoints at the time of testing [28]. Isolates were classified as penicillin susceptible (MIC ≤ 0.064 μg/ml), penicillin intermediate resistant (MIC > 0.064, ≤ 1.0 μg/ml) or penicillin resistant (MIC > 1 μg/ml). Isolates were classified as ceftriaxone susceptible (MIC ≥ 1.0 μg/ml), ceftriaxone intermediate resistant (MIC 2 μg/ml) or ceftriaxone resistant (MIC ≤ 4 μg/ml).

### Pulse field gel electrophoresis

Pulse field gel electrophoresis (PFGE) typing was performed as previously described [29] on a subset of 199 pneumococcal isolates that were available for investigation in May 2003. These isolates were from samples collected between April 1999 and April 2002. Isolates were from 99 children: 53 non-Aboriginal children contributed 97 pneumococci and 46 Aboriginal children contributed 102 pneumococci. A single isolate per sample was typed and the pneumococcal isolates represented a range of serotypes.

### Multilocus sequence typing

To further investigate a multiresistant 6B clone identified by PFGE, multilocus sequence typing (MLST) was performed on a subset of 6B isolates (*n* = 12) using a mass spectrometry based method [30]. Isolates were selected based upon antimicrobial susceptibility profiles with approximately half from Aboriginal and half from non-Aboriginal children. As serotype 19A is commonly associated with serotype replacement following 7vPCV introduction [31], twelve 19A isolates from this study and six 19A isolates collected in Kalgoorlie during 2008 as part of pneumococcal surveillance in Western Australian Aboriginal people [32] were also analysed by MLST.

### Data analysis

Statistical analyses were performed using SPSS (version 15.0 for Windows) and the level of statistical significance was set at *p* < 0.05. The Mann–Whitney *U* test was used to compare the age of Aboriginal and non-Aboriginal children at vaccination and time from vaccination to sample collection. Pearson’s chi square was used to test differences in total vaccine doses received between Aboriginal and non-Aboriginal children. The modified Wald method was used to calculate confidence intervals of proportions using GraphPad (GraphPad Software Inc, USA) and the Z test used to compare proportions.

When investigating the effect of vaccination on carriage, to avoid potential bias related to health-seeking behaviour (e.g. delayed attendance for immunisation), the cohort was restricted to include data from those children who never received 7vPCV during the study period and those who received their first dose of 7vPCV before age 12 months. Among 7vPCV recipients, only samples collected >14 days after 7vPCV administration were considered post-vaccine to allow for the immunologic response to develop. Logistic regression modeling was used to assess the relationship between 7vPCV vaccination and pneumococcal carriage whilst adjusting for age. The models incorporated Generalized Estimating Equations (GEEs) to account for loss of independence due to repeated sampling of individuals. The regression analysis was performed separately for Aboriginal and non-Aboriginal children. Non-typeable pneumococci were excluded from this analysis.

## Results

A total of 506 nasopharyngeal samples were collected from Aboriginal children and 1,045 from non-Aboriginal children. The median number of samples collected was 6 for both Aboriginal and non-Aboriginal children. The median age at first sample collection was 15 days for Aboriginal children and 17 days for non-Aboriginal children. Information on vaccination status is presented in Additional file 1: Table S1. The overall pneumococcal carriage rates (% of samples containing any pneumococcus) were 49.2 % for Aboriginal children and 24.9 % for non-Aboriginal children, with carriage rates increasing with age as previously reported [11]. A total of 547 distinct pneumococcal isolates were identified and subject to further analysis.

### Pneumococcal serotypes

**Fig. 1:**
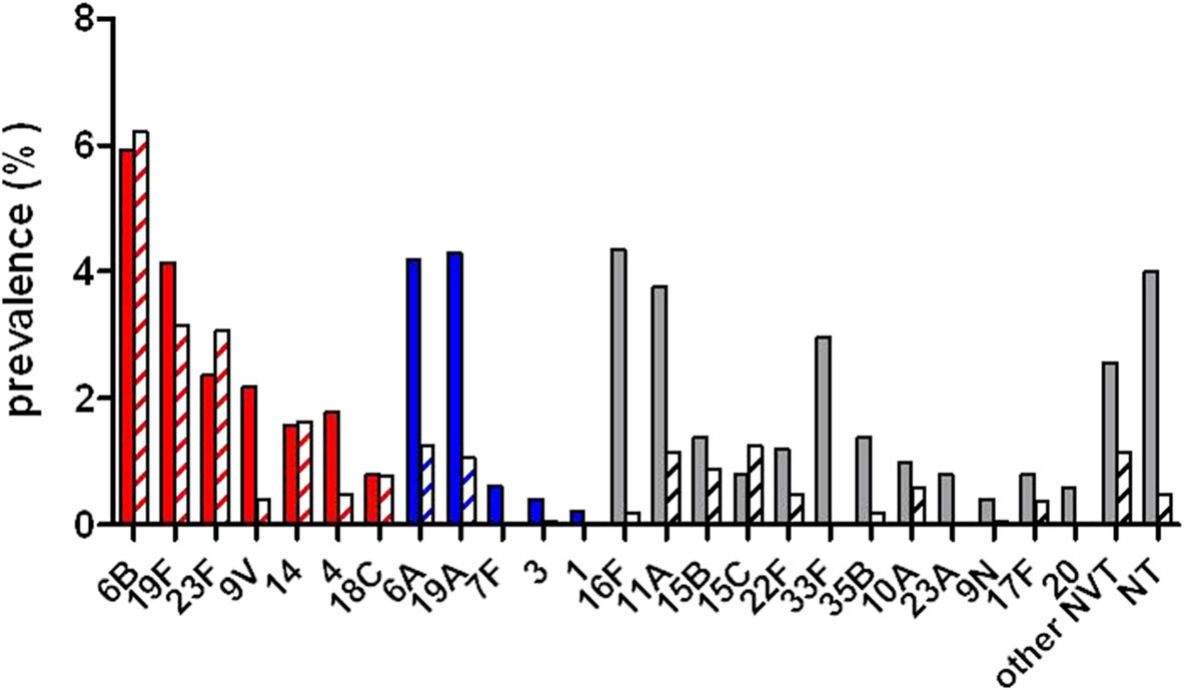
Prevalence (% of samples) of pneumococcal serotypes in carriage samples collected from Aboriginal (solid bars) and non-Aboriginal (hashed bars) study participants (1999–2005). 7vPCV serotypes are shown in red, with the additional serotypes contained in 13vPCV shown in blue and non-vaccine types (NVT) shown in grey. NT = non-typeable

The prevalence (% of samples) of pneumococcal serotypes (7vPCV serotypes, non-7v PCV13 serotypes, and non-vaccine types) in study participants is shown in Fig. 1, with full data available in Additional file 1: Table S2. Overall, 18.8 % (95 % CI 15.6–22.4) of samples from Aboriginal children contained 7vPCV serotypes compared to 15.7 % (95 % CI 13.6–18.0) of samples from non-Aboriginal children. There were no significant differences in the prevalence of 7vPCV between Aboriginal and non-Aboriginal groups when examined in different age groups (<6 months 17 % vs 10 %, 6 - <12 months 21 % vs 23 %, and ≥12 months 22 % in both). There were also no differences in prevalence of the most common serotype, namely 6B, between Aboriginal and non-Aboriginal children in the 3 age groups (<6 months 3-4 %, 6- < 12 months 11 %, ≥12 months 8-10 %). Data for other individual serotypes were too sparse to investigate in different age groups. For the additional six serotypes contained in 13vPCV, rates were 9.7 % (95 % CI 7.4–12.6) from Aboriginal children and 2.7 % (95 % CI 1.8–3.9) from non-Aboriginal children (note that serotype 5 was not detected in this study). Non-vaccine serotypes were more common in samples from Aboriginal children (21.9 % [95 % CI 18.5–25.8]) compared to non-Aboriginal children (7.1 % [95 % CI 5.7–8.8]), with similar patterns in different age groups. A broader range of serotypes was seen in Aboriginal children (33 different serotypes in 506 samples) than in non-Aboriginal children (29 serotypes in 1,045 samples, *p* = 0.0004). Serotypes 6A, 19A, 16F, 11A, 33F, 9V, and non-typeable pneumococci were more commonly found in samples from Aboriginal than non-Aboriginal children. Twenty-five (4.9 %) samples from Aboriginal children yielded multiple pneumococcal serotypes (24 samples with two different serotypes and one sample with three different serotypes). Twelve (1.1 %) samples from non-Aboriginal children produced two different serotypes.

### Antimicrobial susceptibility

A total of 526 pneumococcal isolates from 507 samples were tested for susceptibility to penicillin, erythromycin, tetracycline, cotrimoxazole, chloramphenicol, and ceftriaxone. Rifampicin and vancomycin susceptibility was tested for on 478 and 479 isolates, respectively. All isolates were susceptible to ceftriaxone, rifampicin, and vancomycin. Results for other antimicrobials are summarised in Table 1, with detailed results by serotype shown in Additional file 1: Tables S3 and S4. Overall, 127 (24.1 %) isolates showed reduced susceptibility to penicillin, 73 (13.9 %) to erythromycin, 78 (14.8 %) to tetracycline and 147 (27.9 %) to cotrimoxazole. There were differences in antimicrobial susceptibility between pneumococci isolated from Aboriginal and non-Aboriginal children. All isolates from Aboriginal children were susceptible to chloramphenicol, whereas 13.2 % of isolates from non-Aboriginal children displayed reduced susceptibility. Reduced penicillin susceptibility of serotypes 19F and 19A was more common and that of serotype 6B less common in Aboriginal than in non-Aboriginal children (19F: 70 % vs. 39 %; 19A 64 % vs. 18 %; 6B 21 % vs. 59 %). Cotrimoxazole resistance was present in almost half of the serotype 6B isolates from Aboriginal children and 74 % of those from non-Aboriginal children. Of note, 65 % of non-typeable pneumococci isolated from Aboriginal and non-Aboriginal children had reduced susceptibility to penicillin and 74 % had reduced susceptibility to cotrimoxazole. Overall, 5 % of 503 serotypeable pneumococci were resistant to two antimicrobials and a further 9 % to three antimicrobials. Equivalent figures for 23 non-typeable pneumococci were 30 % and 43 %, respectively.

**Table 1:** Reduced antimicrobial susceptibility of *Streptococcus pneumoniae* in nasopharyngeal samples from Aboriginal and non-Aboriginal children

Isolate type	Number of isolates	% with reduced susceptibility to antimicrobial agent				
		Penicillin^a^	Erythromycin	Tetracycline	Cotrimoxazole	Chloramphenicol
7vPCV types						
Aboriginal	92	31.5	7.6	17.4	35.9	0.0
Non-Aboriginal	162	35.2	26.5	27.2	43.8	21.6
Non-vaccine types						
Aboriginal	151	15.2	4.0	3.3	4.6	0.0
Non-Aboriginal	97	3.1	6.2	3.1	13.4	0.0
Non-typeable						
Aboriginal	18	66.7	50.0	27.8	72.2	0.0
Non-Aboriginal	5	60.0	40.0	60.0	80.0	0.0
All pneumococci						
Aboriginal	261	24.5	8.4	10.7	22.6	0.0
Non-Aboriginal^b^	265	23.8	19.2	18.9	33.2	13.2

^a^Pneumococci showed intermediate resistance to penicillin (MIC 0.065-1.0 μg/mL) except for 1 serotype 23F isolate that showed MIC =1.5 μg/mL

^b^Includes 1 isolate not serotyped

There were 33 isolates (serotypes 6B, *n* = 27; 23F, *n* = 5; 19F, *n* = 1) from 20 non-Aboriginal children, which showed reduced susceptibility to penicillin, tetracycline, erythromycin, cotrimoxazole and chloramphenicol (6.3 % of all isolates, 12.5 % of isolates from non-Aboriginal children). This multi-resistance pattern was not seen in any isolates from Aboriginal children.

### Molecular epidemiology

Using PFGE, 67 different molecular types of *S. pneumoniae* were identified among the 199 isolates examined (Additional file 1: Table S5). Of these, 30 were represented once and 14 types were seen five or more times. All 7vPCV serotypes in this population were quite variable except 18C. Only one molecular type (72) was found in both a vaccine (6B) and a non-vaccine (10A) serotype; these isolates were not from the same child.

MLST was performed on 12 serotype 6B isolates and 18 serotype 19A isolates (6 of which were collected in 2008) (Table 2). The multiresistant 6B clone identified as molecular type 58 by PFGE was revealed to be ST90, a globally distributed clone also known as Spain^6B^-2. Interestingly, ST90 was only found in non-Aboriginal children, whereas ST185, a globally distributed clone known as S. Africa^6B^-8 was isolated from both Aboriginal and non-Aboriginal children.

**Table 2:** Genotypes and antimicrobial resistance of select 6B and 19A isolates

Isolates (*n*)	Ethnicity	Serotype	Antimicrobial resistance^a^	PFGE type^b^	Sequence Type^c^
1	Aboriginal	6B	cotrimoxazole *(I)*, tetracycline *(R)*	59	385
1	Aboriginal	6B	tetracycline *(R)*	ND	385
4	Aboriginal (3), non-Aboriginal (1)	6B	cotrimoxazole *(R)*	60 (2), ND (2)	185 (DLV 385)
1	Aboriginal	6B	cotrimoxazole *(R)*	ND	4276 (DLV 385)
4	Non-Aboriginal	6B	multiresistant	58, ND	90
1	Non-Aboriginal	6B	multiresistant	ND	10265^d+^ (SLV 90)
1	Aboriginal	19A	none	30	199
1	Aboriginal	19A	none	28	199
1	Non-Aboriginal	19A	none	11	199
1	Aboriginal	19A	cotrimoxazole *(R)*	31	876 (SLV 199)
1	Non-Aboriginal	19A	cotrimoxazole *(R)*	ND	10264^d^ (DLV 199)
2	Aboriginal, non-Aboriginal	19A	none	18	172
1	Aboriginal	19A	none	34	10262^d^
2	Aboriginal	19A	none	ND	10263^d^ (SLV 10262)
1	Non-Aboriginal	19A	cotrimoxazole *(R)*	ND	162
4 (3 from 2008)	Aboriginal	19A	cotrimoxazole *(R)*, tetracycline *(R)*	ND	202
3 (from 2008)	Aboriginal	19A	multiresistant	ND	320

^a^ *I* Intermediate, *R* Resistant, *multiresistant* resistant to cotrimoxazole, erythromycin, and tetracycline, and chloramphenicol and/or penicillin

^b^ *ND* not determined

^c^ *SLV* single locus variant, *DLV* double locus variant

^d^ *ST* first reported in this study

The 6B isolates clustered into two broad clonal complexes, one related to ST385 and one related to ST90. These two clonal complexes were evenly distributed throughout the study period. Most of the 19A isolates were related to ST199, although one 19A isolate was ST162, which is mainly associated with serotypes 9V and 19F [33]. Three isolates with distinct PFGE molecular types were all ST199. The six more recent 19A isolates examined belonged to ST202 and ST320. No serotype 6B isolates were identified in 2008 following widespread use of 7vPCV.

### Pneumococcal carriage and vaccination

A higher proportion of Aboriginal than non-Aboriginal children received 7vPCV, they were older than non-Aboriginal children at time of receiving each dose of 7vPCV and there was no difference in time from receiving 7vPCV to sample collection between Aboriginal and non-Aboriginal children (Additional file 1: Table S1). Table 3 shows pneumococcal carriage in unimmunised children and according to number of doses among those who received their first dose of 7vPCV before age 12 months. Serotypes were grouped as 7vPCV and non-7vPCV types for this analysis as numbers were too small to analyse individually for most serotypes. Similarly, children receiving 1 and 2 doses of 7vPCV were grouped due to small numbers. There were 415 samples from Aboriginal children and 1,010 samples from non-Aboriginal children included in this analysis (82 % and 97 % of all study samples, respectively). From this cohort of Aboriginal children, pneumococci were isolated from 105 (40.9 %) samples from unvaccinated children, 44 (57.1 %) samples from those who had received one or two doses of 7vPCV, and 53 (65.4 %) samples from those who had received 3 doses. Equivalent numbers for the non-Aboriginal children were 183 (22.3 %), 33 (37.1 %), and 33 (32.4 %), respectively. Note that the increased pneumococcal carriage with more doses is related to the increase in carriage prevalence with age during the first year of life [11].

**Table 3:** Proportion of samples with 7vPCV or non-7vPCV serotypes in Aboriginal and non-Aboriginal children who received 0, 1–2, or 3 doses of 7vPCV

		Doses			Total
		0	1-2	3	
Aboriginal	Samples (n)	257 (%)^a^	77 (%)	81 (%)	415 (%)
	7vPCV serotypes	46 (17.9)	16 (20.8)	8 (9.9)	70 (16.9)
	Non-7vPCV serotypes	63 (24.5)	27 (35.1)	38 (46.9)	128 (30.8)
Non-Aboriginal	Samples (n)	819 (%)	89 (%)	102 (%)	1010 (%)
	7vPCV serotypes	110 (13.4)	24 (27.0)	18 (17.6)	152 (15.0)
	Non-7vPCV serotypes	72 (8.8)	11 (12.4)	14 (13.7)	97 (9.6)

^a^% = proportion of samples

Note: To avoid potential bias related to health service utilization, data presented here are from children who either never received 7vPCV or received their first dose of 7vPCV before age 12 months. Among 7vPCV recipients, only samples collected >14 days after 7vPCV administration were considered post-vaccine

Some nasopharyngeal aspirates included more than one 7vPCV or non-7vPCV serotypes or both 7vPCV and non-7vPCV serotypes. Non-typeable pneumococci were excluded

A higher proportion of samples from Aboriginal children had non-7vPCV serotypes after receiving 3 doses of vaccine (46.9 %, 95 % CI 36.4–57.7 %) compared with samples from those who were not vaccinated (24.5 %, 95 % CI 19.6–30.1 %), while carriage of 7vPCV serotypes was lower following 3 doses of vaccine than in unvaccinated children (9.9 % [95 % CI 4.9–18.5 %] vs. 17.9 % [95 % CI 13.7–23.1 %]). In contrast, similar proportions of 7vPCV and non-7vPCV pneumococcal serotypes were found in unvaccinated and fully vaccinated non-Aboriginal children. After controlling for age and accounting for repeated sampling, the odds of isolating 7vPCV pneumococcal serotypes in samples from Aboriginal children were about five times less after three doses of vaccine than in samples from unvaccinated children, and the odds of isolating non-7vPCV types were 2.5 times greater (Table 4). Similar results were not seen with samples taken from non-Aboriginal children. However, non-Aboriginal children who received 1–2 doses of 7vPCV had an increased odds of carrying 7vPCV serotypes.

**Table 4:** Odds Ratios for carriage of *S. pneumoniae* serotypes in nasopharyngeal samples by vaccine doses received

Serotype	Dose 1/2 OR^a^ (95 % CI)		Dose 3 OR (95 % CI)	
	Aboriginal	Non-Aboriginal	Aboriginal	Non-Aboriginal
7vPCV serotypes	0.90 (0.43, 1.88)	1.93 (1.13, 3.32)	0.19 (0.08, 0.44)	0.83 (0.41, 1.66)
Non-7vPCV serotypes	1.83 (1.04, 3.20)	1.25 (0.57, 2.74)	2.50 (1.17, 5.35)	1.19 (0.65, 2.17)

^a^OR odds ratio from logistic regression, adjusting for age and age-squared and accounting for repeated sampling of individuals, with unvaccinated as the reference category. Non-typeable pneumococci were excluded

## Discussion

To our knowledge, this is the only study to date comparing the characteristics of pneumococci isolated from the upper respiratory tract in indigenous children with those in non-indigenous children living in the same geographic area, and notably in an arid zone of Australia, for which there is a dearth of any carriage data. In addition to higher carriage rates, the distribution of pneumococcal serotypes was more diverse, and carriage of non-typeable strains and simultaneous carriage of multiple serotypes were more common in Aboriginal than in non-Aboriginal children.

The serotype distribution and antimicrobial susceptibility patterns differed between Aboriginal and non-Aboriginal children. Of note, non-PCV7 serotypes 16F, 19A, 6A, and 33F (known to be replacement serotypes following widespread use of PCVs [34, 35]) were more common in Aboriginal than in non-Aboriginal children. These differences may relate, in part at least, to vaccination status: more Aboriginal children received three doses of 7vPCV, and these fully vaccinated children were less likely to carry 7vPCV serotypes (Table 4), providing evidence of vaccine impact on carriage. However, unvaccinated Aboriginal children had higher carriage rates overall and higher carriage of non-7vPCV serotypes (more serotype diversity) than unvaccinated non-Aboriginal children, indicating some baseline differences in pneumococcal carriage.

Non-Aboriginal children receiving 1 or 2 doses of 7vPCV had increased odds of carrying 7vPCV serotypes when compared to those who had had no 7vPCV. This finding was unexpected and the reasons are unclear. It may relate to temporal variation in carriage of certain serotypes or association between health-seeking behaviour and carriage (i.e. a small proportion of non-Aboriginal parents chose to have their children vaccinated and those who were vaccinated may have been more sickly and more likely to carry pneumococci), or the lack of herd effects in this community as 7vPCV was not included in the national immunization schedule for non-Aboriginal children during the study period. But the small number of non-Aboriginal children in this study who received 7vPCV precludes further analysis.

Generally, reduced susceptibility to antimicrobial agents was observed more often in *S. pneumoniae* carried by non-Aboriginal than Aboriginal children, with all multi-resistant strains isolated from non-Aboriginal children. An exception was serotype 19F which was more commonly resistant when carried by Aboriginal children. The antimicrobial prescription frequency was similar in Aboriginal and non-Aboriginal children in the study, but there are no available data on specific antimicrobial agents [36]. The reasons for the differences in susceptibility to antimicrobial agents are unclear, but may relate to differences in circulating pneumococcal clonal types between the two population groups or potential differences in the classes of antibiotics prescribed.

Carriage of serotype 6B was common in both Aboriginal and non-Aboriginal children, but the multi-resistant clone ST90 was only observed in non-Aboriginal children. A multiple-drug resistant clone of 6B was known to be circulating in Australia from 1988 to 1997 [37]; however, no MLST results are available from this time period so it is not known whether this clone was related to ST90. Reduced susceptibility to antimicrobials was commonly observed in non-typeable isolates from both populations, consistent with reports identifying non-typeable pneumococci as a major reservoir for antimicrobial resistance genes [38, 39].

As expected, the widespread use of PCV in more recent years has altered serotype prevalence in carriage. Surveillance of pneumococcal carriage in Aboriginal people in Western Australia from 2008 to 2011 found a 7 % prevalence in 7vPCV serotypes across varying age groups, with serotypes 19A, 16F and 6C the most common in children under five years old [32]. Further shifts in serotype distribution and a reduction in carriage of 19A have been reported following the switch to PCV13 in 2011 [40]. MLST analysis of serotype 19A isolates suggests that circulating strains may have changed following 7vPCV introduction. Only a small number of isolates was examined, so expanded molecular epidemiological studies would be needed to investigate whether selective pressure following PCV introduction results in clonal expansion, as has been observed in other settings [41].

Limitations of this study include the fact that antiserum to serotype 6C (required to differentiate between 6A and 6C) was not available at the time of sample analysis, so this serotype would not have been detected. It is also likely that simultaneous carriage of multiple serotypes was underestimated [42]. Non-typeable pneumococci were not subject to genetic analysis, therefore could represent both isolates not producing capsule and “true” non-typeable pneumococci that lack capsule synthesis genes. More sophisticated serotyping methodologies are now available [43]. Antibiotic susceptibility data were not examined according to vaccine status, due to small numbers within each dosing group.

An overall reduction in invasive pneumococcal disease (IPD) and a reduction in IPD caused by 7vPCV serotypes have been observed in the post-PCV years in Australia, although IPD due to non-vaccine serotypes has increased [13, 44]. The switch to PCV13 in 2011 has led to further reductions in IPD, particularly in children under five years of age [45]. Although a gap still remains, the disparity in pneumonia hospitalisations between Australian Aboriginal and non-Aboriginal children has declined over time, likely due in part to PCV introduction [46].

## Conclusions

This study identified important differences in pneumococcal serotypes, genotypes, and antimicrobial susceptibility between Aboriginal and non-Aboriginal children living in the same region before widespread 7vPCV use. Ongoing surveillance of pneumococcal carriage is warranted to monitor potential increases in antimicrobial resistance and changes in serotype distribution that could impact on pneumococcal disease and health outcomes, and to help in the continual development of effective vaccines. Data reported here provide a useful baseline comparator, as well as insight into some of the differences in *S. pneumoniae* carried by Aboriginal and non-Aboriginal children in Australia.

## Additional file

Additional file 1: Table S1. 7vPCV vaccination characteristics of the study participants. **Table S2.** Prevalences of pneumococcal serotypes found in the respiratory tract of study participants 1999–2005. **Table S3.** Reduced antimicrobial susceptibility of *Streptococcus pneumoniae* in nasopharyngeal samples from Aboriginal children reported by serotype. **Table S4.** Reduced antimicrobial susceptibility of *Streptococcus pneumoniae* in nasopharyngeal samples from non-Aboriginal children reported by serotype. **Table S5.** The molecular type by pulse-field gel electrophoresis (PFGE) of 199 *S. pneumoniae* isolates reported by serotype. (DOCX 26 kb)

## Abbreviations

23vPPV: 23-valent pneumococcal polysaccharide vaccine
7vPCV: 7-valent pneumococcal conjugate vaccine
CI: confidence interval
IPD: invasive pneumococcal disease
MIC: minimum inhibitory concentration
MLST: multilocus sequence typing
NPA: nasopharyngeal aspirate
OM: otitis media
OR: odds ratio
PCV: pneumococcal conjugate vaccine
PFGE: pulse field gel electrophoresis
