# Supplementary material for: Aboriginal and non-Aboriginal children in Western Australia carry different serotypes of pneumococci with different antimicrobial susceptibility profiles
Source: Pneumonia (Nathan). 2016 Sep 5;8:15. doi: 10.1186/s41479-016-0015-9 (PMC5471808; doi:10.1186/s41479-016-0015-9)
Supplement: Additional file 1: Table S1. — 7vPCV vaccination characteristics of the study participants. Table S2. Prevalences of pneumococcal serotypes found in the respiratory tract of study participants 1999–2005. Table S3. Reduced antimicrobial susceptibility of Streptococcus pneumoniae in nasopharyngeal samples from Aboriginal children reported by serotype. Table S4. Reduced antimicrobial susceptibility of Streptococcus pneumoniae in nasopharyngeal samples from non-Aboriginal children reported by serotype. Table S5. The molecular type by pulse-field gel electrophoresis (PFGE) of 199 S. pneumoniae isolates reported by serotype. (DOCX 26 kb) [file 41479_2016_15_MOESM1_ESM.docx]

**Supplementary Table 1.** 7vPCV vaccination characteristics of the study participants.

| **Vaccination characteristic** | **Aboriginal children** | **Non-Aboriginal children** | **P value** |
| --- | --- | --- | --- |
| Total 7vPCV doses received | No. (%) children | No. (%) children |  |
| 0 | 32 (32.0) | 123 (68.3) | < 0.001 |
| 1 | 9 (9.0) | 4 (2.2) | 0.009 |
| 2 | 11 (11.0) | 8 (4.4) | 0.035 |
| 3 | 48 (48.0) | 45 (25.0) | < 0.001 |
| Median age at 7vPCV vaccination | Months (No. children) | Months (No. children) |  |
| Dose 1 | 4.1 (68) | 3.7 (57) | 0.017 |
| Dose 2 | 6.4 (59) | 5.2 (53) | 0.016 |
| Dose 3 | 9.1 (48) | 7.7 (45) | 0.011 |
| Median time from 7vPCV vaccination to sample collection | Days (No. samples) | Days (No samples) |  |
| Dose 1 | 49 (55) | 54 (44) | 0.741 |
| Dose 2 | 61 (42) | 49 (47) | 0.462 |
| Dose 3 | 309 (87) | 231 (114) | 0.157 |

**Supplementary Table 2.** Prevalences of pneumococcal serotypes found in the respiratory tract of study participants 1999 - 2005.

| **Serotype** | **Aboriginal(number samples = 506)** | | **Non-Aboriginal**  **(number samples = 1045)** | |
| --- | --- | --- | --- | --- |
| **7vPCV type** | **n** | **%** | **n** | **%** |
| 4 | 9 | 1.8 | 5 | 0.5 |
| 6B | 30 | 5.9 | 65 | 6.2 |
| 9V | 11 | 2.2 | 4 | 0.4 |
| 14 | 8 | 1.6 | 17 | 1.6 |
| 18C | 4 | 0.8 | 8 | 0.8 |
| 19F | 21 | 4.2 | 33 | 3.2 |
| 23F | 12 | 2.4 | 32 | 3.1 |
| *Total* | *95* | *18.8* | *164* | *15.7* |
| **PCV13/ non-PCV7 type** |  |  |  |  |
| 1 | 1 | 0.2 | - |  |
| 3 | 2 | 0.4 | 4 | 0.4 |
| 6A | 21 | 4.2 | 13 | 1.2 |
| 7F | 3 | 0.6 | - |  |
| 19A | 22 | 4.3 | 11 | 1.0 |
| *Total* | *49* | *9.7* | *28* | *2.7* |
| **Non-PCV type** |  |  |  |  |
| 7C | 2 | 0.4 | 1 | 0.1 |
| 8 | - |  | 1 | 0.1 |
| 9A | 1 | 0.2 | 2 | 0.2 |
| 9N | 2 | 0.4 | 6 | 0.6 |
| 10A | 5 | 1.0 | 6 | 0.6 |
| 11A | 19 | 3.8 | 12 | 1.2 |
| 13 | 2 | 0.4 | - |  |
| 15A | - |  | 1 | 0.1 |
| 15B | 7 | 1.4 | 9 | 0.9 |
| 15C | 4 | 0.8 | 13 | 1.2 |
| 16F | 22 | 4.3 | 2 | 0.2 |
| 17F | 4 | 0.8 | 4 | 0.4 |
| 18A | 1 | 0.2 | - |  |
| 18B | - |  | 1 | 0.1 |
| 20 | 3 | 0.6 | - |  |
| 21 | 1 | 0.2 | - |  |
| 22A | - |  | 3 | 0.3 |
| 22F | 6 | 1.2 | 5 | 0.5 |
| 23A | 4 | 0.8 | 3 | 0.3 |
| 23B | 2 | 0.4 | 1 | 0.1 |
| 29 | 2 | 0.4 | - |  |
| 33F | 15 | 3.0 | - |  |
| 34 | 1 | 0.2 | 1 | 0.1 |
| 35B | 7 | 1.4 | 2 | 0.2 |
| 38 | 1 | 0.2 | 1 | 0.1 |
| *Total* | *111* | *21.9* | *74* | *7.1* |
| Nontypeable pneumococci | 20 | 4.0 | 5 | 0.5 |

**Supplementary Table 3.** Reduced antimicrobial susceptibility of *Streptococcus pneumoniae* in nasopharyngeal samples from Aboriginal children reported by serotype.

|  |  | % with reduced susceptibility to antimicrobial agent | | | |
| --- | --- | --- | --- | --- | --- |
| **Isolate type^a^** | **Number of isolates** | **Penicillin^b^** | **Erythromycin** | **Tetracycline** | **Cotrimoxazole** |
| *All 7vPCV types* | *92* | *31.5* | *7.6* | *17.4* | *35.9* |
| 6B | 29 | 20.7 | 3.4 | 37.9 | 48.3 |
| 9V | 11 | 45.5 | 0.0 | 0.0 | 27.3 |
| 14 | 8 | 0.0 | 12.5 | 0.0 | 0.0 |
| 18C | 3 | 0.0 | 0.0 | 0.0 | 66.7 |
| 19F | 20 | 70.0 | 25.0 | 25.0 | 30.0 |
| 23F | 12 | 33.3 | 0.0 | 0.0 | 66.7 |
|  |  |  |  |  |  |
| *All Non-vaccine types* | *151* | *15.2* | *4.0* | *3.3* | *4.6* |
| 6A (PCV13) | 18 | 0.0 | 0.0 | 0.0 | 11.1 |
| 19A (PCV13) | 22 | 63.6 | 0.0 | 9.1 | 18.2 |
| 10A | 5 | 20.0 | 20.0 | 20.0 | 40.0 |
| 9A | 1 | 100.0 | 0.0 | 0.0 | 0.0 |
| 9N | 2 | 0.0 | 50.0 | 50.0 | 0.0 |
| 11A | 18 | 0.0 | 0.0 | 0.0 | 22.2 |
| 15B | 7 | 14.3 | 0.0 | 0.0 | 0.0 |
| 16F | 21 | 14.3 | 4.8 | 4.8 | 0.0 |
| 22F | 6 | 16.7 | 16.7 | 16.7 | 16.7 |
| 29 | 2 | 0.0 | 100.0 | 0.0 | 0.0 |
| 34 | 1 | 0.0 | 0.0 | 100.0 | 0.0 |
| 35B | 7 | 28.6 | 0.0 | 0.0 | 0.0 |
|  |  |  |  |  |  |
| *Non-typeable* | *18* | *66.7* | *50.0* | *27.8* | *72.2* |
|  |  |  |  |  |  |
| All pneumococci tested | 261 | 24.5 | 8.4 | 10.7 | 22.6 |

^a^All isolates were susceptible to ceftriaxone and chloramphenicol. Only those serotypes which showed reduced susceptibility to at least one antimicrobial agent are listed separately.

^b^Pneumococci showed intermediate resistance to penicillin (MIC 0.065-1.0µg/mL)

**Supplementary Table 4.** Reduced antimicrobial susceptibility of *Streptococcus pneumoniae* in nasopharyngeal samples from non-Aboriginal children reported by serotype.

|  |  | % with reduced susceptibility to antimicrobial agent | | | | |
| --- | --- | --- | --- | --- | --- | --- |
| Isolate type^a^ | Number of isolates | Penicillin^b^ | Erythromycin | Tetracycline | Cotrimoxazole | Chloramphenicol |
| *All 7vPCV types* | *162* | *35.2* | *26.5* | *27.2* | *43.8* | *21.6* |
| 6B | 65 | 58.5 | 44.6 | 43.1 | 73.8 | 43.1 |
| 14 | 17 | 0.0 | 11.8 | 0.0 | 0.0 | 0.0 |
| 18C | 8 | 0.0 | 0.0 | 0.0 | 100.0 | 0.0 |
| 19F | 33 | 39.4 | 18.2 | 33.3 | 27.3 | 3.0 |
| 23F | 30 | 20.0 | 20.0 | 16.7 | 20.0 | 20.0 |
|  |  |  |  |  |  |  |
| *All Non-vaccine types* | *97* | *3.1* | *6.2* | *3.1* | *13.4* | *0.0* |
| 6A | 13 | 0.0 | 7.7 | 0.0 | 7.7 | 0.0 |
| 19A | 11 | 18.2 | 0.0 | 0.0 | 18.2 | 0.0 |
| 10A | 6 | 0.0 | 16.7 | 16.7 | 83.3 | 0.0 |
| 15A | 1 | 0.0 | 100.0 | 0.0 | 100.0 | 0.0 |
| 15B | 6 | 0.0 | 0.0 | 0.0 | 33.3 | 0.0 |
| 15C | 12 | 8.3 | 16.7 | 0.0 | 8.3 | 0.0 |
| 16F | 2 | 0.0 | 50.0 | 50.0 | 0.0 | 0.0 |
| 18B | 1 | 0.0 | 0.0 | 100.0 | 0.0 | 0.0 |
| 22A | 3 | 0.0 | 0.0 | 0.0 | 33.3 | 0.0 |
|  |  |  |  |  |  |  |
| *Non-typeable* | *5* | *60.0* | *40.0* | *60.0* | *80.0* | *0.0* |
|  |  |  |  |  |  |  |
| All pneumococci tested ^c^ | 265 | 23.8 | 19.2 | 18.9 | 33.2 | 13.2 |

^a^ All isolates were susceptible to ceftriaxone. Only individual serotypes which showed reduced susceptibility to at least one antimicrobial agent are listed separately.

^b^ Pneumococci showed intermediate resistance to penicillin (MIC 0.065-1.0µg/mL) except for 1 serotype 23F which showed MIC =1.5 μg/mL

^c^ Includes 1 isolate not serotyped

**Supplementary Table 5.** The molecular type by pulse-field gel electrophoresis (PFGE) of 199 *S. pneumoniae* isolates reported by serotype.

| **Serotype** | **Molecular type*** | **No. isolates** | **No. molecular types** |
| --- | --- | --- | --- |
| *7vPCV types* | |  |  |
| 4 | 39 (5), 45 | 6 | 2 |
| 6B | 19 (2), 53 (4), 54 (2), 55, 56, 58 (12), 59 (5), 60 (7), 61 (2), 62 (2), 63, 72 (2) | 41 | 12 |
| 9V | 44, 46 (2), 48 (5) | 8 | 3 |
| 14 | 5, 20, 51(3), 52 | 6 | 4 |
| 18C | 57 (7) | 7 | 1 |
| 19F | 10, 12, 13, 16, 21 (2), 32 (3), 34, 35 (5), 49, 68 (4) | 20 | 11 |
| 23F | NT (2), 36 (3), 37, 38, 39, 40 (4) | 12 | 6 |
| *PCV13 types* | |  |  |
| 3 | 4 | 1 | 1 |
| 6A | NT, 5, 6, 7 (3), 8, 9, 64 (2), 67 (2), 71 | 13 | 9 |
| 19A | 11, 14, 17 (5), 18 (5), 28, 30, 31 (2), 34 (2), 50 | 19 | 9 |
| Non-vaccine types | |  |  |
| 7C | 2 | 1 | 1 |
| 9N | 47 (5) | 5 | 1 |
| 10A | 72 (4) | 4 | 1 |
| 11A | 26 (12), 27 (3) | 15 | 2 |
| 15B | NT (2), 23, 29, 30 (4) | 8 | 4 |
| 15C | 30 (7) | 7 | 1 |
| 16F | 41 (5), 42 (5), 43 (2) | 12 | 3 |
| 17F | 73 | 1 | 1 |
| 18B | 57 | 1 | 1 |
| 22A | 69 | 1 | 1 |
| 22F | 25 (4) | 4 | 1 |
| 23A | NT | 1 | 1 |
| 29 | 22 | 1 | 1 |
| 33F | 24 (4) | 4 | 1 |
| 35B | 15 | 1 | 1 |

*The number of isolates belonging to each molecular type is shown in parentheses. NT = not typeable by PFGE.
